# Human pericardium graft in the management of bleb's complication performed in childhood: a case report

**DOI:** 10.1186/1471-2415-11-27

**Published:** 2011-09-20

**Authors:** Dimitris Papaconstantinou, Ilias Georgalas, Sergios Taliantzis, Chrysanthi Koutsandrea, Ioannis Ladas, Gerassimos Georgopoulos

**Affiliations:** 1 Department of Glaucoma, Athens University Medical School, Athens, Greece

**Keywords:** late bleb leakage, human pericardium, bleb revision, pediatric glaucoma

## Abstract

**Background:**

To report a case with hypotony due to late leakage of the filtering bleb performed during childhood and treated surgically using human pericardium graft.

**Case Presentation:**

A man with hypotony related to bleb's leakage in his right eye was presented. During his childhood trabeculectomy was performed to manage ocular hypertension due to pediatric glaucoma. Biomicroscopy revealed choroidal tissue incarcerated in the sclerectomy under the conjunctiva. Bleb revision was performed. Human pericardium graft was used to cover the sclerectomy and a new bleb with controlled outflow was created. The intraocular pressure (IOP) and Seidel test represent the main outcomes. Intraoperative and postoperative complications were recorded. Fifteen days postoperatively the IOP was of 7 mmHg and the bleb seemed to filter properly. Five months later the IOP was 9 mmHg and no complications were noticed. During the follow up time, the Seidel test was negative.

**Conclusion:**

We used human pericardium graft with no complications in a case of bleb leakage performed for pediatric glaucoma.

## Background

Congenital glaucoma is the major cause of blindness in children, despite its low incidence (1:10,000 births) [1]. Several surgical procedures have been advocated to restore the aqueous humor pathway and to lower the elevated intraocular pressure (IOP) [2].

Trabeculectomy has been proposed as a valuable procedure, to facilitate the outflow of aqueous humor, but is not very effective in congenital glaucoma. This feature has been attributed to the inherent tendency that young tissues have toward excessive scarring [3]. The intraoperative use of adjunctive antimetabolites, like 5-fluorouracil (5FU) and mitomycin C (MMC), has been proposed to enhance the final outcomes of trabeculectomy, in pediatric glaucoma patients [4].

Antimetabolites, especially MMC, increment the risk of vision-threatening complications such as cystic and blebs leakage, ocular hypotony, maculopathies, choroidal hemorrhage and infections like blebities and endophthalmities. Late bleb leakage occurs more often in thin, avascular blebs and correlates with full thickness surgical procedures and use of antimetabolites [5].

We report our surgical approach to control the hypotony in a case with leaking filtering bleb, which occurred years after the trabeculectomy.

## Case presentation

**Figure 1:**
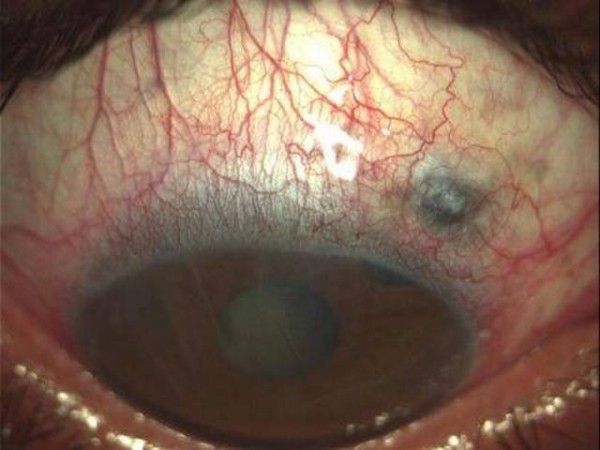
**Preoperative: Notice the choroidal tissue under the conjunctiva**.

A young man of 30 years old was presented to our clinic, claiming blurring of vision in his right eye. Best corrected visual acuity (BCVA) was of light perception. Slit lamp examination revealed corneal decompensation with Descemet membrane folds, swallowing of the anterior chamber and hypotony associated with choroidal detachment, detected in the B-mode ultrasound examination. The IOP could not be measured (IOP < 0 mmHg). There was a filtering bleb on the upper and temporal side of his right eye. Under the conjunctiva we noticed the absence of a scleral flap. Instead, choroidal tissue could be seen (Figure 1). The Seidel test was negative, because of the choroidal tissue incarcerated into the slerectomy. His left eye was without relevant clinical problems. The patient referred to a trabeculectomy performed twenty years ago during his childhood. No other information about the surgical procedure or the eventual intraoperative use of antimetabolites was available.

We decided to correct surgically the filtering bleb and the correlated hypotony, following the guidelines of the declaration of Helsinki and obtaining approve by the Ethics Committee of G.N.A. "G. Geminnatas" University Athens hospital. A 7-0 silk corneal traction suture was used to fixate and infraduct the globe, increasing exposure of the surgical site. A fornix-based conjunctival flap on the superotemporal side of the right eye was performed and the conjunctiva and tenon's capsule were separated from the sclera. Once the sclera was uncovered, we confirmed the absence of the scleral flap and the presence of choroidal tissue incarcerated in the sclerectomy. Human pericardium graft (sterile human tissue allograft, New World Medical, Rancho Cucamonga, California, USA) was used to cover the sclerectomy. Four 10-0 nylon sutures were placed to the graft in its four angles, over the sclerectomy and 8-0 Vicryl sutures were used to suture the conjunctiva above the graft. A new filtering bleb was created, with human pericardium graft, instead of a scleral flap, covering the sclerectomy. No antimetabolites were used intraoperatively.

Fifteen days postoperatively the BCVA was of light perception, the intraocular pressure (IOP) was of 7 mmHg, the cornea had Descemet membrane folds and the anterior chamber was narrow. The bleb filtered properly, the pericardium graft could be seen under the conjunctiva and the Seidel test was negative.

**Figure 2:**
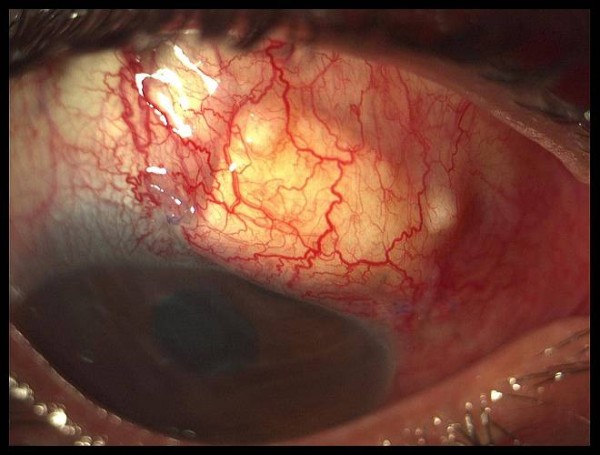
**Postoperative: Five months after surgery**.

Five months later no complications were recorded. The new bleb was working properly and the Seidel test was negative. The patient had BCVA of light perception because of end state glaucomatous optic disk atrophy, the IOP was of 9 mmHg and less Descemet membrane folds and deeper depth of the anterior chamber could be seen (Figure 2).

## Conclusions

Late bleb leakage (LBL) occurs months to years after glaucoma filtering surgery (GFS), with an incidence of about 1 to 10% [6].

Our patient probably underwent a full thickness trabeculectomy, without a scleral flap protection [7]. We don't know if antifibrotic agents were used during or after surgery, and also if the scleral flap was performed as usual and has been melted or became inadequate through years. We decided to use human pericardium graft sutured over the sclerectomy, as a substitute of the missing scleral tissue. A new bleb, covered with conjunctiva, was easily performed with no complications. The use of human pericardium over donor scleral graft presents several advantages, because it is available commercially, has a long shelf life and is very easy to handle intraoperatively.

We have not found references concerning the use of human pericardium graft for the repair of late onset leakage of a bleb performed for pediatric glaucoma.

This method does not require a special learning curve and it creates a new system of controlled humor aqueous' outflow.

## Consent

Written informed consent was obtained from the patient for publication of this case report and any accompanying images.

## Competing interests

The authors declare that they have no competing interests.

## Authors' contributions

DP, IG and GG were in charge of the medical care of the patient and performed the different surgeries. CK, IL and ST were responsible for the photographs and the literature review. DP, IG and ST wrote the manuscript. GG, CK and IL reviewed it, drafted it critically and provided helpful comments. All authors read and approved the final manuscript.

## Pre-publication history

The pre-publication history for this paper can be accessed here:

http://www.biomedcentral.com/1471-2415/11/27/prepub
